# Changes in Skin Microcirculation Resulting from Vibration Therapy in Women with Cellulite

**DOI:** 10.3390/ijerph19063385

**Published:** 2022-03-13

**Authors:** Anna Piotrowska, Olga Czerwińska-Ledwig, Małgorzata Stefańska, Tomasz Pałka, Marcin Maciejczyk, Przemysław Bujas, Marek Bawelski, Tomasz Ridan, Małgorzata Żychowska, Ewa Sadowska-Krępa, Agnieszka Dębiec-Bąk

**Affiliations:** 1 Institute for Basic Sciences, Faculty of Physiotherapy, University of Physical Education, 31-571 Krakow, Poland; anna.piotrowska@awf.krakow.pl (A.P.); olga.czerwinska@awf.krakow.pl (O.C.-L.); 2 Department of Physiotherapy in Motor Dysfunction, Faculty of Physiotherapy, Wroclaw University of Health and Sport Sciences, 51-612 Wroclaw, Poland; malgorzata.stefanska@awf.wroc.pl; 3 Department of Physiology and Biochemistry, Faculty of Physical Education and Sport, University of Physical Education, 31-571 Krakow, Poland; tomasz.palka@awf.krakow.pl (T.P.); marcin.maciejczyk@awf.krakow.pl (M.M.); marek.bawelski@awf.krakow.pl (M.B.); 4 Institute of Sports, University of Physical Education, 31-571 Krakow, Poland; przemyslaw.bujas@awf.krakow.pl; 5 Department of Kinesitherapy, University of Physical Education, 31-571 Krakow, Poland; tomasz.ridan@awf.krakow.pl; 6 Department of Biological Foundations of Physical Culture, Kazimierz Wielki University, 85-091 Bydgoszcz, Poland; zychowska.m@gmail.com; 7 Institute of Sport Sciences, The Jerzy Kukuczka Academy of Physical Education, 40-065 Katowice, Poland; e.sadowska-krepa@awf.katowice.pl

**Keywords:** thermovision, vibrotherapy, skin temperature, cellulite, physical therapy, skin microcirculation

## Abstract

Background: Cellulite is a cosmetic defect that affects over 80% of post-pubertal women. One of its pathomechanisms involves microvascular dysfunction. It has been suggested that vibration is a physical stimulus that may improve circulation in the skin and muscles. The aim of this study was to evaluate the effect of local vibration on cutaneous microcirculation and on eliminating the symptoms of cellulite in women. Methods: A total of 57 healthy women with at least grade 1 cellulite were recruited and divided into four groups differing by treatment time (30′ or 60′) and position (sitting or lying) during the vibration treatments. Participants took part in 15 vibrotherapy sessions. Body composition, selected circumferences, cellulite grade, and thermographic images of buttocks and thighs were recorded. Results: Significant changes in skin temperature were observed in both studied areas after the first and last treatments in each group. A significant decrease in cellulite grade was observed after a series of treatments. The strongest effects were observed for the sitting position with a treatment time of 60 min. Conclusion: Vibration treatment improves microcirculation in cellulite-affected areas. Over time, no adaptation was observed, and subsequent treatments maintained the beneficial effects. Extending the treatment time increased its influence on the microcirculation in the skin.

## 1. Background

Cellulite is a cosmetic defect that affects approximately 80–90% of post-pubertal women. In men, this defect is rare (about 2%) and occurs as a symptom accompanying diseases related to the secretion of androgens, such as Klinefelter’s syndrome, hypogonadism, post-castration states, or from adverse reactions to estrogen pharmacotherapy [1]. The difference in the frequency of occurrence between the sexes is a consequence of hormone activity (in women, the role of the correct balance and cyclical changes in the concentration of progestins and estrogens is emphasized), differences in the histological structure of the skin and subcutaneous adipose tissue, and differences in the number of adipocytes. Excess subcutaneous fat tissue penetrates deep into the dermis, giving the characteristic appearance of “orange peel” when pressed upon [2].

In reducing cellulite, non-invasive, non-surgical treatments (topically applied creams and massage, including Chinese cupping massage), or instrumental treatments (extracorporeal shock wave therapy and radiofrequency-, laser-, and light-based-device therapies) are used. Some invasive methods are also used, e.g., liposuction and injection methods (e.g., the administration of collagenase or the filling of structural changes with fillers of various types) [1,3].

Vibrotherapy is one of the methods for minimizing or reducing the visibility of cellulite. It can be used in the form of monotherapy [4,5,6,7] or combined with other treatment forms, such as non-focused ultrasound with Aussie current and low-level laser therapy [8,9]. The medical application of this physical stimulus is a relatively old idea. Regardless of the form of propagation of the vibration stimulus (whole body vibration or locally applied vibration), several mechanisms of action are indicated: (1) the tonic vibration reflex [10]; (2) blocking signals in the posterior horns of the spinal cord, which cause pain relief (even after a single exposure to vibration) [11]; and (3) effects on blood vessels. An analysis of the literature by Games et al. [12] suggested that the acute effect of whole body vibration (WBV) is related to the increase in peripheral blood flow but not to changes in levels of muscle oxygenation. The parameters describing the vibration (frequency, amplitude, and intensity) are of great importance in modulating this effect. Vibration also generates an increase in blood flow through the skin [12], which may involve a medical (e.g., improved drainage and transport of metabolites, cytokines, and enzymes released from cells) as well as cosmetic or aesthetic aspect.

Some authors assume that the main cause of cellulite lies in microcirculatory alterations because in the area affected by cellulite, the blood flow is 35% lower than in nonaffected regions [1,13,14]. The improvement of microcirculation is the mechanism of action in many active cosmetic ingredients used in cosmetic preparations [1]. Therefore, we supposed that vibration, which is a recognized factor influencing blood vessels and blood and lymph circulation, would favorably influence cellulite-affected tissues. We expected that vibrotherapy would improve skin microcirculation and its aesthetic effects would be visible as the elimination of cellulite changes in the thighs and buttocks. An important problem in the methodology of research on the effectiveness of cosmetic and esthetic medical treatments is to identify a tool that allows for an objective evaluation of the obtained results. This is now a substantial issue in the assessment of these kinds of therapies and their effectiveness in the assumed goals of therapy [15].

In recent years, in the diagnosis of various cosmetic defects, an increasing use of thermal imaging has been observed. This technique allows for the assessment of the effectiveness of treatment processes and control of skin condition. Medical thermography is used as a tool for assessing the effectiveness of esthetic and physical therapy procedures in which selected physical factors (e.g., extremely low temperatures, ultrasonic waves, thermal waves, and vibrations) are used. In such therapeutic methods, the parameters of the stimuli are usually selected empirically or on the basis of the size of the sensory or motor reaction, and thus the observation and registration of reactions taking place during and after the procedure is particularly important. Thermovision is a useful tool in such treatments, allowing for a one-time assessment of the reaction of the stimulated area in the microcirculation; it also provides the opportunity to monitor and evaluate treatments performed in a series [16,17]. Thermography is a completely safe and non-invasive method that allows for the determination of temperature distribution on the skin surface, and it can show differences in temperature distribution which could be affected by the physiological state of the examined body areas [17]. The operating principles of thermographic devices are related to the detection and registration of infrared radiation (IR) sent by the examined object. In medicine, thermographic systems operating in a specific range of waveslengths (2–5 or 8–12 μm) are used [17]. Thermography can be an additional diagnostic tool that allows a more detailed analysis of the body’s physiological reactions to the stimulus used. Thanks to the above-described advantages, thermovision imaging is widely used in medicine and also in the interpretation of the impact of thermal and vibration stimuli in therapy or biological regeneration in the human body.

The aim of this work was to indirectly assess the impact of vibration therapy on the cutaneous microcirculation of the buttocks and thighs and to determine the effectiveness of the therapy through the reduction of cellulite in women.

## 2. Materials and Methods

### 2.1. Ethical Declaration

The study design received a positive evaluation from the Bioethical Committee of PMWSZ in Opole (Poland), which approved the study (no. KB/56/N02/2019). None of the procedures performed in this study violated the provisions of the Helsinki Declaration. All activities were undertaken after obtaining written consent from project participants in accordance with the procedures of the Central Scientific and Research Laboratory (PN-EN ISO 9001: 2015: PW-08606-19) of AWF Kraków. Participants were informed of the purpose and research methods of the study and were given a chance to view the results and withdraw from participation in the project without any consequences, at any stage, without giving any reason.

### 2.2. Study Group

In this study, 57 healthy women were recruited. The following inclusion criteria were established: female sex with the presence of cellulite of a minimum intensity of at least grade 1 according to the Nürnberger and Müller scale, and no contraindications for local vibrotherapy treatment, i.e., history of cancer, cardiovascular disease, thrombosis, surgical intervention involving the musculoskeletal system, acute back pain, or advanced diabetes; current presence of pacemaker, acute inflammation, recovery period after endoprosthesis of the hip or knee joint, or infectious diseases; presence of headache, dizziness, or nausea occurring during or after treatment [2].

**Figure 1:**
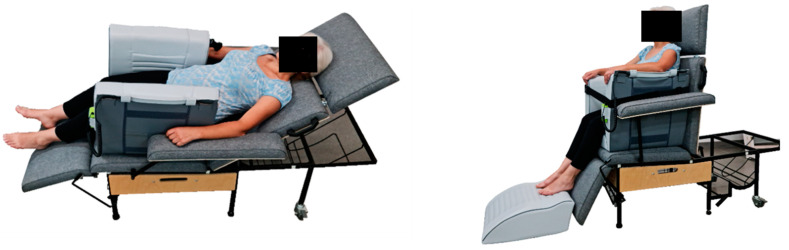
The positions of the participants during vibration treatments (authors’ own material).

In the recruitment process, the women were asked to complete a short questionnaire on smoking and the use of hormonal contraception. Then, the estimation of body composition and the circumference measurement of selected body parts were performed, and grades of cellulite were determined. The subjects were randomly assigned to 1 of 4 groups differing in time (30 or 60 min) and the position (sitting or lying) used during the vibration treatments (Figure 1). Additionally, all study participants started the treatments in the same phase of the menstrual cycle (follicular phase). Vibrotherapy sessions were held at different times of the day so as not to interfere with the professional duties of the women participating in the project. Detailed characteristics of the 4 study groups are presented in Table 1.

### 2.3. Study Protocol

Recruited women who fulfilled the inclusion criteria participated in a series of 15 vibrotherapy treatments applied in a sitting (S30 and S60) or lying (L30 and L60) position. The treatments were performed once a day, 5 days a week (Monday to Friday) for 3 consecutive weeks.

Body weight was measured before the first and last treatments. The first and last treatments of the series were performed in a fasted state, and the subjects were asked to empty their bladders before the sessions. Before and after the first treatment, and before and after the last treatment, body composition and the selected body circumferences were determined. Thermographic images were taken at the same time points. Each participant had four thermograms taken during participation in the study: before the first treatment (1), after the first treatment (2), before the last treatment (3), and after the last treatment (4).

### 2.4. Research Methods

#### 2.4.1. Body Composition and Circumferences

A JAWON MEDICAL (Korea) analyzer was used to estimate body composition (through the bioelectric impedance method) according to the standards of Dehghan and Merchant [18]. The analysis was performed in the morning on an empty stomach. The following parameters were determined: body weight (BM), fat-free mass (FFM), total fat (TF), and total body water (TW).

Body circumferences were measured using an anthropometric tape. The measurements were always performed by the same researcher on the non-dominant arm and leg. Hip circumference (HC) was measured at the widest point. The remaining circumferences (WC, waist circumference; and TC, thigh circumference) were measured according to the NHANES guidelines [19].

#### 2.4.2. Thermographic Research

After acclimatization, in accordance with the standards of the European Thermology Association (http://eurothermology.org/ (accessed on 5 May 2019), before the vibrotherapy treatment and 10 min after the end of the procedure, the examined women were photographed using a Thermo GEAR G120EX camera with a microbolometer detector: resolution 320 × 240 pixels, thermal sensitivity 0.04 at 30 °C, and accuracy ±2 °C (NEC Avio Infrared Technologies Co., Ltd., Tokyo, Japan).

The thermograms were taken in a room with constant thermal conditions (22 °C) and controlled air humidity (45–50%), with low, fixed lighting at a stable distance. Obtained images included the backs of the thighs and buttocks. The distance between the subject and the camera lens was 120 cm. Thermograms were analyzed with InfReC NS9500 Standard software (NEC Avio Infrared Technologies Co., Ltd., Tokyo, Japan).

In the assessment of the thermograms, contours of the shape of the anatomical areas affected by cellulite were adjusted individually for each participant, and for all 4 images taken of each participant, a personalized outline (shape) was used. In the designated areas, the maximum temperature (T_max_), the minimum temperature (T_min,_), and the average temperature (T_ave_) were determined. For each analysis, it was assumed that the determination of the minimum and maximum temperatures should be performed in such a way that they covered 99.7% of the pixels comprising the thermogram.

#### 2.4.3. Cellulite Grading Assessment

The grade of cellulite was assessed by the palpation-visual method according to the Nürnberger and Müller scale [2]. The assessments were always performed by the same researcher, qualifying the condition of the skin according to the following scheme: grade 0—healthy skin, no unevenness even when the skin was pressed; grade 1—smooth skin, both in the standing and lying positions, unevenness becomes visible only after pressing the skin; grade 2—skin irregularities visible only in the standing position, smooth skin in the lying position; grade 3—uneven skin visible both in the standing and lying positions.

#### 2.4.4. Intervention

Vibration treatments were performed with use of the hip module of the MMT device (Vitberg, Nowy Sącz, Poland) in which the oscillatory-cycloidal vibrations were generated by a motor operating in the range of 18–39 Hz. The symmetrical work of the side modules in the cushions placed on the sides of the body was assumed. A single operation cycle of the device, in accordance with the manufacturer’s settings, lasted 30 min and included vibration with variable parameters in accordance with ISO standards (PN-ISO 5805). The groups undergoing the treatments in the 60-min protocol received two cycles of 30 min each. During the procedure, the participants were wearing light sports clothes, without footwear.

### 2.5. Statistical Analysis

The Saphiro-Wilk test was used to verify the distribution of all measured parameters, which in most cases was found to be close to normal. Descriptive statistics were calculated (means and standard deviations; SD). In order to assess the statistical significance of the differences between the results obtained in the 4 measurements in all study groups, taking into account the time and position of the treatment and the grade of cellulite, ANOVA with repeated measurements was used. Nominal variables were analyzed using the contingency tables and the Chi^2^ test. Correlation between variables was estimated by Pearson’s correlation coefficient r. The Eta^2^ parameter was used to assess the effect size for ANOVA. The Eta^2^ interpretation values were as follows: <0.01 = small effect, <0.06 = intermediate effect, and <0.14 = large effect [20]. Cramer’s V coefficient was a measure of the effect for the Chi^2^ test. Interpretation of Cramer’s V was as follows: <0.3 = small effect, <0.5 = moderate effect, and >0.5 = large effect [21]. The significance level was *p* < 0.05. All analyses were performed using the statistical program STATISTICA (version 13.1).

## 3. Results

**Figure 2:**
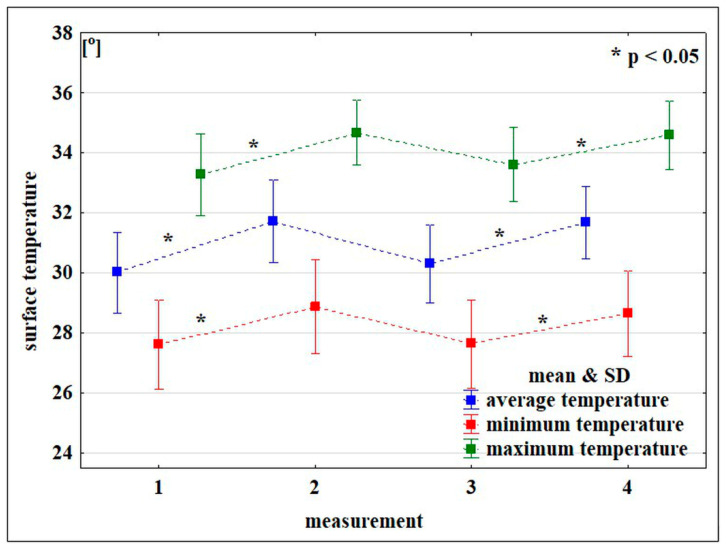
The surface temperature of the buttocks and thighs recorded in subsequent measurements during vibration therapy.

**Figure 3:**
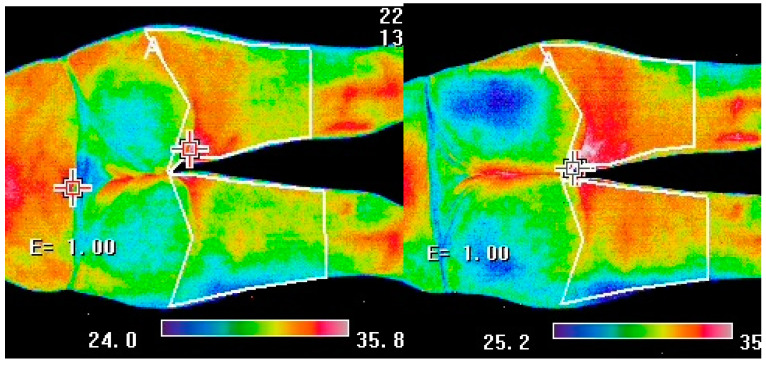
Exemplary thermograms taken before (on the **left**) and 10 min after a vibrotherapy session (on the **right**).

The mean, minimum, and maximum surface temperatures of the buttocks and thighs, recorded after the vibration treatment, were found to be statistically significantly higher compared with the measurements performed before the procedure. Significant differences for the temperature changes were found for both the first (measurements 1 vs. 2) and the last treatments (measurements 3 vs. 4). However, no significant changes were found between measurements 1 (performed before the 1st treatment) and 3 (performed before the last treatment). The conducted analysis showed significantly lower values of T_min_ recorded in the sitting position compared with the lying position. There were no significant effects for the duration of the procedure and the grade of cellulite for the measured temperature values (Table 2 and Table 3, Figure 2). Exemplary thermograms taken from one participant before and after treatment are shown in Figure 3.

The performed analysis did not confirm any significant changes in the levels of total fat and total water estimated in subsequent measurements. On the other hand, lower values of the TF levels were observed in the group of women subjected to 60-min procedures compared with the group subjected to 30-min procedures. Statistically significant lower levels of TF and higher TW levels in women with grade 1 cellulite compared with the group with grade 2 were also found (Table 2 and Table 4).

The performed analysis did not confirm any significant changes in hip and thigh circumferences during subsequent measurements. On the other hand, a statistically significant decrease in WC was observed between the 1st and the 4th measurements. Significantly lower circumferences of all measured segments were also documented in the group of women with grade 1 cellulite compared to the group with grade 2 (Table 2 and Table 5).

Vibration therapy significantly reduced ellulite grade. In 51% of participants who exhibited grade 2 cellulite before the therapy, it decreased to grade 1. Accordingly, among those with grade 1 cellulite in the initial examination, it decreased to grade 0 in 54.5% of cases (Table 6).

Correlations between the surface temperature of the buttock and thigh areas subjected to vibrotherapy and selected body composition parameters and circumferences were investigated. Statistically significant correlations were confirmed only in the group of women with grade 1 cellulite. For the first measurement performed before the start of the treatments, a negative, moderate correlation was observed between TF and a moderate positive correlation between TW and the mean and minimum temperatures of the analyzed body regions. T_min_ measured in the buttock and thigh areas showed a significant negative correlation with WC in the 3rd and 4th measurements and with HC in the 4th. A moderate negative correlation of T_min_ with TC in the study was also confirmed (1st and 3rd) (Table 7).

## 4. Discussion

The temperature distribution visible in thermal images of the surface of the skin depends on several factors: the amount of skin blood flow, ambient temperature, sweat evaporation, diffusion of water vapor through the skin, heat loss during respiration, type of clothing, convection, and the amount of radiated energy [17]. Important factors which should also be considered for obtaining objective and correctly interpreted results for thermal imaging are regular menstrual cycles, having a stable body weight for at least three months, and maintaining regular sports habits [22]. The time of day and even the consumption of a hot drink before the thermogram is taken may also be important in the analysis of the dynamics of changes in the surface temperature of the studied body area [22]. All the above-mentioned information means that under controlled conditions and defined standards, thermal imaging can be a useful tool in assessing skin temperature [17]. In this study, we took into account as many factors that could influence thermal imaging results as possible for the purpose of showing the actual influence of vibrotherapy on the temperature of the skin affected by cellulite changes.

Thermographic analysis has already been used in research studies to assess the severity of cellulite changes. However, its form and the devices used may vary and are not standardized. Dedicated, liquid crystal cliches that allow for a quick assessment of temperature distribution on the skin of the thighs are available, but this is a contact method with relatively low resolution of the obtained image, and the evaluation of the results is subjective [23]. The non-contact method with the use of a thermal imaging camera is characterized by high resolution, and images are analyzed with the use of dedicated software, so it is a better objective tool for assessing the effectiveness of anti-cellulite therapies that do not affect temperature distribution in studied body areas and is widely used in scientific research [6,22,24].

In our study, after the analysis of the thermograms, it was shown that women with grades 1 and 2 cellulite did not differ in terms of skin temperature in the assessed areas. We also noted a significant increase in temperature after a single vibration treatment. This effect was observed after both the first and last treatments in the series. The effect of treatment position and the duration of a single procedure were not observed.

As a result of the analysis of the obtained thermograms, it was possible to show that only in women with grade 1 cellulite were there correlations between temperature and measured anthropometric and body-composition parameters. The fact that in women with grade 2 cellulite no such relationship was found, in our opinion, may be indirectly related to a greater impairment of microcirculation in the cutaneous vascular and lymphatic bed associated with the pathomechanism of cellulite formation [1]. According to a presumed microcirculation mechanism, the formation of cellulite lesions is related to microcirculation disorders which cause local accumulation of cytokines and other molecules that induce changes in the function and metabolism of local tissues. Fat cells increase their volume, which causes an increase in pressure exerted on the blood vessels; this implies further disturbances in adipose-tissue metabolism. The blood and lymph flow become obstructed, which leads to fluid stasis in the vessels and exudation. This manifests on the surface of the skin in the form of numerous cavities and bumps characteristic of cellulite changes [14]. It is worth emphasizing that in this project, only the thermograms of women with grades 1 and 2 cellulite were assessed. These are the most common forms of cellulite in women. However, we cannot assume that for women with more severe forms of cellulite, the results would be similar—a further study should be carried out for this purpose.

Thermal imaging of the skin enables the documenting of general changes in the skin, including the causes of cellulite [25]. Monitoring skin temperature gives information concerning blood flow in the skin and the tissues lying directly underneath [26,27]. In previously published papers, the relationship between skin temperature and cellulite was emphasized. In the work of Nkengne et al. [22], based on the observation of a limited number of subjects photographed in various conditions, the thermovision method was used to assess cellulite. The authors considered all four types of cellulite. Image analysis allowed the identification of parameters describing general skin temperature, temperature homogeneity, and visual contrast between hot and cold areas in the thermograms. A study by Migasiewicz et al. [24] showed that mean skin temperature and skin temperature homogeneity are associated with cellulite. The thermal images clearly show that cellulite increases the number of hot and cold spots present on affected skin. The authors cited earlier suggested that hot spots may be related to swelling caused by leakage of fluid from damaged blood vessels, while cooler spots may be related to blood stasis [22]. The present study’s authors’ own research did not indicate the influence of the grade of cellulite on average skin temperature. However, only women with grades 1 and 2 cellulite were examined, which limits the insight into the most common but also less severe form of this aesthetic defect. On the other hand, it was possible to observe that only in women with grade 1 cellulite, thermographically evaluated temperatures correlate with the parameters of body composition and selected body circumferences. This fact shows that these parameters (especially TW and TF) can modulate the accuracy of the thermographic measurements used to assess the effectiveness of cosmetic treatments. It was shown that adipose tissue can influence average skin temperature values. Neves at al. observed that women have a lower skin temperature than men, which is mainly related to their higher percentage of BF [28]. Higher fat percentages in specific anatomical segments were related to lower skin temperatures, especially in the posterior thighs, lower legs, and arms [29]. Our research shows that not only are gender, body fat content, and BF content in individual body segments important for the distribution of skin surface temperature, but specific features of surface tissues, such as cellulite, are also important here.

Average skin temperature analysis has limited use in monitoring changes in the cellulite grade of subjects with low grades of cellulite. However, this indicated a tendency to improve the flow in the vascular bed of the skin of the thighs and buttocks in women undergoing a three-week vibrotherapy treatment series. Such effects were observed for women participating in the procedures in a sitting position (which is probably related to the distribution of pressure forces and better adhesion of tissues to the device generating the vibration stimulus) for 60 min (which is probably the effect of the dose-dependence of the obtained effect). It was also noted that the tendency to increase the average skin temperature occurred only in women with grade 1 cellulite. This appears to be due to the improvements visible in the thermographic imaging of women with this cellulite grade.

We showed that vibrotherapy is an effective form of reducing the severity of cellulite changes. Reductions in cellulite grade in the examined women were observed. The vibrational stimulus may act through several mechanisms (tonic vibration reflex, increased circulation in the vascular bed of the skin and subcutaneous tissues, friction, and local heat generation) and it depends, inter alia, on the form of stimulus propagation in the tissues. The basic differences in this case concern WBV and vibration applied locally [30].

The results of Milanese et al. [31] suggest that WBV sessions improve body composition and may be an effective addition to lifestyle recommendations for obese women. However, a large meta-analysis of available research studies on the effects of WBV on body composition did not report optimistic results and showed that only some specific populations can achieve benefits from this form of treatment [32].

Although vibration exercise has gained popularity for weight loss and body shaping, it cannot replace aerobic exercise in terms of energy costs. However, it seems that, similar to resistance training, vibration exercises have a positive effect on blood flow [33], which can increase the metabolic rate in the relevant areas and can improve lean body mass and muscle strength without significantly affecting selected skin fold thickness [34]. The form of vibrotherapy used in this study provides a more delicate stimulus than WBV, and it also has fewer contraindications. Our research hypothesis did not assume that vibration would change body composition. However, it allowed for the improvement of aesthetics and for reduced visibility of cellulite.

Our research clearly indicated that when analyzing individual anthropometric and body-composition parameters, the grade of cellulite is a differentiating factor.

The study of the influence of WBV on the symptoms of cellulite was the aim of the study by Cristovam et al. [4]. This controlled, clinical study was conducted on 42 women with cellulite in the buttocks area. The qualified women were divided into two groups: those taking part in vibration treatments and the control group. At the beginning and at the end of the first and the last (10th) WBV sessions, the surface temperature of the skin, perimetry of the buttock area, body contour analysis, aesthetic improvement analysis, and satisfaction with the therapy were assessed. In the thermographic analysis, a significant increase in the surface temperature of the buttock skin was obtained in the vibration group, which, despite the differences in the form of stimulus propagation, is consistent with our observations. There were no differences within groups or between groups in perimetry and body contour analysis. The vibration group achieved a higher percentage of improvement in the evaluation of esthetics and satisfaction. These results give a mixed picture of the effectiveness of WBV as a monotherapy for cellulite reduction.

WBV could also be combined with other forms of anti-cellulite therapy. The aim of the study by Savoia et al. [9] was the evaluation of the effectiveness of the use of low-level laser therapy (635 nm and 0.040 W) in combination with WBV (4–25 Hz) in patients with local obesity and cellulite. A total of 33 people (18–64 years in age) were included in the study. A significant reduction in fat thickness was observed after six weeks of therapy, and the authors emphasized that the proposed combined therapy was not only effective but also safe. In the work of Canela et al. [8], a combination of non-focused ultrasound and Aussie current with WBV was used. The authors compared the effect of these treatments alone and also when combined with vibration stimulus. The study involved 20 healthy women aged 20–40 years with grade 2 or grade 3 cellulite. Each participant underwent 10 WBV sessions over a 5-week period. In the evaluation of the results, standardized photography, skin ultrasound examination, cutometer measurement, and questionnaires were used. It was shown that this therapy combined with WBV gives more favorable results for most of the measured factors.

As indicated previously, there are two forms of use for vibration stimulus: use with a platform (WBV, described above) and local application. The second type of treatment has also been successfully used to eliminate cellulite. Studies in which local vibration was used included hand-held devices or other devices generating vibration. The aim of the study by Sadowski et al. [35] was the evaluation of the reduction in volume and the improvement in the visual appearance of cellulite after applying local vibration for 24 weeks. In this study, 40 healthy volunteers used a hand-held device that generated vibrations and applied it to the outer and back sides of the thighs. After the initial 12 weeks of continuous massage, the next 12-week phase allowed for the assessment of the dynamics of the disappearance of the treatment effect. The results emphasized that cellulite was significantly reduced after 12 weeks of local vibrotherapy. However, it returned to baseline shortly after treatment discontinuation, while cellulite remained reduced in the subgroup which continued the use of the device. The results of Sadowski et al. [35] show that the effect of vibration on cellulite-affected tissues was present during the application of a series of treatments but disappeared quickly. This may be convergent with our results. Each treatment had a significant effect on skin temperature, but we did not observe a long-term effect from a series of treatments. The research by Sadowski et al. [35] and ours show that devices generating local vibrations reduce cellulite and that continuous use of vibration massage is beneficial for alleviating the visible signs of cellulite.

Different results were obtained by Pilch et al. [6]. The aim of this pilot study was to evaluate the condition and temperature of cellulite-affected skin after local vibration treatment in young women. 10 healthy women (21.5 ± 1.5 years) with grades 1 or 2 cellulite participated in this study. The subjects underwent a four-week series of 20 vibration treatments performed five times a week, 60 min each. Before and after the first and last interventions, thermograms were taken. Complete remission of cellulite after the applied vibration procedure was demonstrated in 40% of respondents. Among the remaining 60% with grade 1 cellulite, an improvement in the condition of the skin was observed, and the grade of cellulite decreased. These results from the study by Pilch et al. are completely consistent with the results presented in this study. The differences, however, appear in the results of the thermogram analysis. The average skin temperature on the side of the thigh as well as on the back of the thigh and buttocks increased significantly after both the first and the last interventions. This is a confirmation of our present results. In the discussed work of Pilch et al. [6], after a series of treatments, however, a statistically significant increase in the mean skin temperature in the lateral area of the thigh was observed, which was not shown in the present study. These differences may be caused by two factors: the group in the study by Pilch was younger and the women in the present project underwent a longer, four-week series of vibrotherapy treatments. As indicated in our study, the action of vibration is dose-dependent regarding time range (the observed differences between the action of 30- and 60-min treatments). However, the dose-dependence of the action of the vibrations is not manifested in all its effects [7]. In this project, the series of treatments used allowed for the reduction of the waist circumference and significant changes in WHR (waist to hip ratio) and WHtR (waist to height ratio) regardless of the time of a single treatment (30 or 60 min). Research shows that the influence of vibrations on circulation is dose dependent. These observations made it possible to distinguish between the safe vibration stimuli used in physiotherapy and biological regeneration and harmful stimuli. The adverse effect of vibrations on the human body is associated with impaired circulation [36]. Safe vibration frequency and amplitude ranges acceptable for therapy have the opposite effect and improve tissue circulation. It was indicated that this is associated with the release of nitric oxide and the increased activity of the enzyme responsible for its synthesis in situ, nitric oxide synthase [37,38,39]. Available research demonstrates that the effect of vibration on blood vessels has a multifaceted mechanism. The repeated use of the vibration stimulus affects the condition of small blood vessels in the skin. As indicated by Pilch et al. [5], vibration applied for a period of four weeks affects the dermatoscopical image of the blood vessels of the skin. This may, on the one hand, improve tissue drainage and, on the other hand, improve the aesthetic appearance of the skin.

## 5. Conclusions

Vibration treatment causes changes in the skin temperature of the thighs and buttocks of women with cellulite, which supports the improvement of skin microcirculation in the studied areas. This effect is not subject to the phenomenon of adaptation. Both the first and the last vibration procedures resulted in a significant increase in the assessed temperature values. After a series of treatments, a significant decrease in the grade of cellulite was noted, regardless of the selected treatment time and body position, but the strongest effects were observed for the sitting position with a treatment time of 60 min.

## Figures and Tables

**Table 1:** Characteristics of the study groups.

Group		All Participants	S30 *n* = 15	L30 *n* = 13	S60 *n* = 15	L60 *n* = 14	ANOVA/Chi^2^ *p* Value
age (year)	mean	22.84	22.47	23.92	21.67	23.50	0.6825
	SD	5.34	4.76	7.18	3.39	5.91	
body height (cm)	mean	166.42	164.87	165.69	165.87	169.36	0.1471
	SD	5.62	4.07	5.99	6.15	5.58	
body mass (kg)	mean	62.28	60.05	63.58	60.73	65.11	0.3354
	SD	8.48	6.96	9.67	8.19	8.76	
BMI (kg/m^2^)	mean	22.28	22.06	22.48	22.00	22.69	0.8322
	SD	2.28	2.17	2.38	1.88	2.81	
smoking yes/no	*n*	9/48	4/11	1/12	2/13	2/12	0.5585
contraception yes/no	*n*	21/36	7/8	3/10	6/9	5/9	0.6253

S: sitting position; L: lying position; 30 and 60: treatment times, in minutes; *p* < 0.05.

**Table 2:** Analysis of the statistical significance of differences between the surface temperature of the buttocks and thighs, selected body composition parameters, and circumferences measured in repeated measurements during 15-treatment vibration therapy (taking into account the position and duration of the procedure and the grade of cellulite).

	*p* Value of ANOVA				Partial Eta^2^ for Measurement Number	Partial Eta^2^ for Grade of Cellulite
	Measurement Number	Treatment Time	Treatment Position	Grade of Cellulite		
T_ave_ (°C)	<0.001 *	0.667	0.0712	0.287	0.55	0.03
T_min_ (°C)	<0.001 *	0.553	0.0162 *	0.100	0.31	0.06
T_max_ (°C)	<0.001 *	0.791	0.2613	0.445	0.45	0.01
TF (%)	0.218	0.024 *	0.1437	0.040 *	0.03	0.08
TW (%)	0.173	0.125	0.4679	0.007 *	0.03	0.14
WC (cm)	0.010 *	0.871	0.7438	0.007 *	0.08	0.14
HC (cm)	0.111	0.586	0.4714	<0.001 *	0.04	0.40
TC [cm)	0.932	0.450	0.9367	<0.001 *	0.01	0.40

T_ave_—average temperature; T_min_—minimum temperature, T_max_—maximum temperature; TF—total fat; TW—total water; WC—waist circumference; HC—hip circumference; TC—thigh circumference; * *p* < 0.05.

**Table 3:** The average, minimum, and maximum values of the surface temperatures of the buttocks and thighs recorded during subsequent measurements during vibration therapy.

Temperature (°C)	S30	L30	S60	L60
	Mean ± SD	Mean ± SD	Mean ± SD	Mean ± SD
T_ave_ 1	29.49 ± 1.84	30.44 ± 1.07	29.74 ± 0.88	30.45 ± 1.23
T_ave_ 2	31.62 ± 1.80	32.14 ± 1.46	31.20 ± 0.80	32.03 ± 1.20
T_ave_ 3	29.84 ± 1.43	29.96 ± 1.55	30.45 ± 1.06	30.86 ± 0.97
T_ave_ 4	31.13 ± 1.22	31.69 ± 1.18	31.84 ± 1.34	32.05 ± 1.02
T_min_ 1	27.05 ± 1.71	27.90 ± 1.26	27.32 ± 0.95	28.26 ± 1.70
T_min_ 2	28.76 ± 1.87	29.24 ± 1.67	28.07 ± 0.91	29.51 ± 1.47
T_min_ 3	27.15 ± 1.81	27.38 ± 1.58	27.69 ± 1.20	28.27 ± 1.16
T_min_ 4	28.07 ± 1.36	28.64 ± 1.51	28.67 ± 1.42	29.22 ± 1.34
T_max_ 1	32.95 ± 1.88	33.52 ± 0.86	32.93 ± 0.99	33.74 ± 1.31
T_max_ 2	34.69 ± 1.39	35.03 ± 1.15	34.25 ± 0.60	34.78 ± 1.03
T_max_ 3	33.36 ± 1.42	33.18 ± 1.31	33.86 ± 1.06	33.91 ± 1.10
T_max_ 4	34.13 ± 1.22	34.47 ± 1.08	34.95 ± 1.01	34.79 ± 1.16

Group: S30—sitting, 30 min; S60—sitting, 60 min; L30—lying, 30 min; L60—lying, 60 min. T_ave_—average temperature; T_min_—minimum temperature, T_max_—maximum temperature; 1, 2, 3, 4—measurement number.

**Table 4:** Estimated total body fat and total body water percentages recorded during consecutive measurements during vibration therapy.

Temperature (%)	S30	L30	S60	L60
	Mean ± SD	Mean ± SD	Mean ± SD	Mean ± SD
TF 1	24.92 ± 3.58	24.63 ± 4.44	23.59 ± 4.16	21.84 ± 3.83
TF 2	25.45 ± 3.94	24.98 ± 4.65	23.99 ± 3.87	22.45 ± 3.83
TF 3	25.20 ± 4.01	25.15 ± 3.00	23.57 ± 0.17	21.11 ± 3.74
TF 4	26.03 ± 4.06	25.48 ± 2.90	24.45 ± 4.11	21.71 ± 0.61
TW 1	56.14 ± 3.00	55.30 ± 3.67	56.13 ± 2.92	56.68 ± 3.56
TW 2	55.45 ± 3.03	54.95 ± 3.85	55.79 ± 2.70	56.75 ± 4.14
TW 3	55.54 ± 2.89	54.81 ± 2.94	56.33 ± 2.89	57.41 ± 3.26
TW 4	54.66 ± 3.01	54.58 ± 2.85	55.62 ± 2.92	56.79 ± 3.16

Group: S30—sitting, 30 min; S60—sitting, 60 min; L30—lying, 30 min; L60—lying, 60 min. TF—total fat; TW—total water; 1, 2, 3, 4—measurement number.

**Table 5:** Waist-, hip-, and thigh-circumference values measured during consecutive time points during vibration therapy.

Temperature (cm)	S30	L30	S60	L60
	Mean ± SD	Mean ± SD	Mean ± SD	Mean ± SD
WC 1	71.16 ± 6.03	72.99 ± 5.12	72.17 ± 5.0	72.61 ± 6.87
WC 2	71.17 ± 5.61	72.61 ± 5.01	71.95 ± 5.27	72.39 ± 7.31
WC 3	71.31 ± 5.99	72.24 ± 6.38	70.73 ± 5.28	71.25 ± 6.77
WC 4	71.15 ± 5.37	71.81 ± 5.69	70.77 ± 5.39	71.24 ± 6.86
HC 1	96.03 ± 7.19	98.42 ± 6.82	97.83 ± 6.83	98.32 ± 6.29
HC 2	96.15 ± 6.96	98.12 ± 6.87	98.63 ± 6.62	97.96 ± 6.12
HC 3	98.14 ± 5.45	98.92 ± 6.21	99.25 ± 5.27	97.42 ± 5.72
HC 4	98.51 ± 5.19	99.12 ± 6.03	99.03 ± 5.30	97.14 ± 5.76
TC 1	56.13 ± 4.60	57.24 ± 4.29	57.05 ± 3.99	57.56 ± 4.51
TC 2	56.10 ± 4.00	56.94 ± 4.07	57.04 ± 4.20	57.79 ± 4.44
TC 3	56.09 ± 4.66	57.32 ± 4.15	56.88 ± 3.67	57.31 ± 4.10
TC 4	56.33 ± 4.39	57.69 ± 4.21	57.23 ± 3.98	57.36 ± 4.52

Group: S30—sitting, 30 min; S60—sitting, 60 min; L30—lying, 30 min; L60—lying, 60 min. WC—waist circumference; HC—hip circumference; TC—thigh circumference; 1, 2, 3, 4—measurement number.

**Table 6:** Observed cellulite grade: before vibration therapy (measurement 1) and after therapy (measurement 2).

		Measurement 2			*p* Value of Chi^2^ Test	Crammer’s V
		Grade 0 Cellulite *n* = 12 (21.1%)	Grade 1 Cellulite *n* = 28 (48.1%)	Grade 2 Cellulite *n* = 17 (29.8%)		
**Measurement 1**	Grade 1 Cellulite *n* = 22 (38.6%)	54.55%	45.45%	0.00%	<0.0001 *	0.72
	Grade 2 Cellulite *n* = 35 (61.4%)	0.00%	51.43%	48.57%		

Measurement 1—before the first vibration treatment; measurement 2—before the last vibration treatment (after 14 treatments); * *p* < 0.05.

**Table 7:** The values of the Pearson correlation r coefficient calculated between the values of the surface temperature of the buttocks and thighs and selected parameters of body composition and circumferences in women with grade 1 cellulite.

Grade 1 Cellulite		TF (%)	TW (%)	WC (cm)	HC (cm)	TC (cm)
T_ave_ (°C)	1	−0.50 *	0.44 *	N	N	−0.50 *
	2	N	N	N	N	N
	3	N	N	N	N	N
	4	N	N	N	N	N
T_min_ (°C)	1	−0.51 *	0.44 *	N	N	−0.46 *
	2	N	N	N	N	N
	3	N	N	−0.46 *	N	−0.50 *
	4	N	N	−0.45 *	−0.49 *	N
T_max_ (°C)	1	N	N	N	N	N
	2	N	N	N	N	N
	3	N	N	N	N	N
	4	N	N	N	N	N

N—correlation not statistically significant; T_ave_—average temperature; T_min_—minimum temperature, T_max_—maximum temperature; TF—total fat; TW—total water; WC—waist circumference; HC—hip circumference; TC—thigh circumference; 1, 2, 3, 4—measurement number; * *p* < 0.05.

## Data Availability

Data available on request.
